# Impact of Tetrazolium Ionic Liquid Thermal Decomposition in Solvothermal Reaction on the Remarkable Photocatalytic Properties of TiO_2_ Particles

**DOI:** 10.3390/nano9050744

**Published:** 2019-05-15

**Authors:** Marta Paszkiewicz-Gawron, Anna Gołąbiewska, Anna Pancielejko, Wojciech Lisowski, Julia Zwara, Monika Paszkiewicz, Adriana Zaleska-Medynska, Justyna Łuczak

**Affiliations:** 1 Department of Environmental Technology, Faculty of Chemistry, University of Gdansk, Wita Stwosza 63, 80-308 Gdansk, Poland; m.paszkiewicz-gawron@ug.edu.pl (M.P.-G.); anna.golabiewska@ug.edu.pl (A.G.); julia.zwara@phdstud.ug.edu.pl (J.Z.); adriana.zaleska-medynska@ug.edu.pl (A.Z.-M.); 2 Department of Process Engineering and Chemical Technology, Chemical Faculty, Gdansk University of Technology, Narutowicza 11/12, 80-233 Gdansk, Poland; anna.prochownik92@gmail.com; 3 Institute of Physical Chemistry, Polish Academy of Sciences, Kasprzaka 44, 01-224 Warsaw, Poland; wlisowski@ichf.edu.pl; 4 Department of Environmental Analysis, Faculty of Chemistry, University of Gdansk, ul. Wita Stwosza 63, 80-308 Gdansk, Poland; monika.paszkiewicz@ug.edu.pl

**Keywords:** ionic liquids, visible light photoactivity, TiO_2_ microparticles, ionic liquid thermal decomposition, solvothermal reaction time

## Abstract

Ionic liquids (ILs) could serve as a structuring agent, a solvent, or a source of dopant during solvothermal synthesis of semiconductors particles. To understand the role of IL during formation of TiO_2_ particles, it is necessary to study the stability of this IL in solvothermal synthesis conditions, as well as studying the surface properties of formed TiO_2_ particles. In view of this, the effect of the 2,3,5-triphenyltetrazolium chloride IL ([TPTZ][Cl]) thermal decomposition during the solvothermal reaction and IL content in the reaction system on photoactivity of TiO_2_ microparticles has been systematically investigated. The samples obtained by using [TPTZ][Cl] exhibited remarkable photocatalytic properties in phenol degradation reaction under visible light. HPLC analysis of the solvothermal reaction medium and X-ray photoelectron spectroscopy (XPS) analysis of TiO_2_ particles revealed that [TPTZ][Cl] was decomposed completely and was incorporated into the TiO_2_ lattice. Generally, increasing the reaction time (1, 4, 12, and 24 h) promoted the TiO_2_ microspheres formation, as well as raising the visible light-induced photocatalytic activity of the photocatalysts. Longer reaction time was also accompanied by an increase in the efficiency of 2,3,5-triphenyltetrazolium chloride decomposition. The properties of the photocatalysts were investigated by means of UV-VIS diffuse reflectance spectroscopy (DRS), BET surface area measurements, scanning electron microscopy (SEM), X-ray powder diffraction (XRD) analysis, and XPS.

## 1. Introduction

Ionic liquids (ILs) are organic salts composed of an organic cation and an organic/inorganic anion, which are characterized by thermal stability, negligibly low vapor pressure, high conductivity and polarity, good dissolving properties and a melting point below 100 °C [1]. Due to their unique properties, these salts find application in versatile directions, e.g., electrochemistry [2], biomedicine [3], pharmacy [4], catalysis [5], and photocatalysis [6,7,8], which are relatively new directions of research.

As an important semiconductor material, TiO_2_ has been widely used as the photocatalyst because of its chemical and biological inertness, high stability against photocorrosion, non-toxicity, low cost, and excellent degradation of organic pollutants [9,10,11]. However, practical applications of the TiO_2_ are still quite limited, mainly due to the low quantum efficiency and the broad bandgap responding only to the UV light. In these contexts, the main challenge of the photocatalysis is improvement of its efficiency in the visible irradiation range. The most common ways to improve the photocatalytic activity of TiO_2_ are: (i) sensitization [12]; (ii) doping by metal or non-metal elements [13]; (iii) surface modifications [14]; or (iv) coupling of wide-band-gap semiconductors with narrow-band-gap semiconductors [15]. In recent times, also ILs are used for preparation of TiO_2_ with enhanced photocatalytic properties. According to the literature, 1-butyl-3-methylimidazolium [BMIM] has been the most popularly used and widely investigated cation of ILs. The 1-butyl-3-methylimidazolium chloride ([BMIM][Cl]) [16], 1-butyl-3-methylimidazolium tetrafluoroborate ([BMIM][BF_4_]) [16,17,18,19,20], 1-butyl-3-methylimidazolium hydroxide ([BMIM][OH]) [21], 1-butyl-3-methylimidazolium bromide ([BMIM][Br]) [7], butyl-3-methylimidazolium octylsulfonate ([BMIM][OctSO_4_]) [7], and 1-butyl-3-methylimidazolium hexafluorophosphate ([BMIM][PF_6_]) [7] were used as active substances to modify TiO_2_ to enhance the photocatalytic activity under visible light irradiation. The less used and popularized ILs in heterogeneous photocatalysis can be mentioned as following: 1-benzyl-3-methylimidazolium chloride ([BenMIM][Cl]), 1-butylpyridinium chloride ([BPy][Cl]), 1-butyl-1-metylpyrrolidinium chloride ([BMPyr][Cl]), tetrabutylammonium chloride ([TBA][Cl]) [22], 1-methyl-3-tetradecylimidazolium chloride ([TDMIM][Cl]) [23], 1-vinyl-3-propylimidazolium iodide ([VPIM][I]) [24], 1-hexadecane-3-methylimidazolium bromide ([C_16_MIM][Br]) [25], and 1-Ethyl-3-methylimidazolium chloride ([C_2_MIM][Cl]) [26].

Based on the above literature, it can be summarized that the increased photocatalytic activity of ILs-TiO_2_ under visible irradiation is associated mainly with: (i) doping by nonmetal elements constituting ILs (such as C, F, P, and B) [17,18,19,22], (ii) surface complex charge transfer (CT) [7], (iii) favoring oxygen vacancies and Ti^3+^ species formation during synthesis [16], and (iv) affected transport of photogenerated charges [8,27,28]. Nevertheless, the mechanism of TPTZ–TiO_2_ excitation is strictly dependent on the decomposition temperature of ILs cation and also on the preparation route of photocatalyst. Some physical and structural properties of photocatalyst, i.e., specific surface area and density of crystalline defects, can be controlled by the synthesis procedure. In this regard, ILs can be used as a solvent, template or structure-directing agent in formation of TiO_2_ particles [6,29]. Additionally, it was revealed that the mechanism of photoexcitation under visible spectrum (λ > 420 nm) for samples modified with ILs may occur in different ways, depending on the degree of IL thermal decomposition [22]. When IL cation is relatively thermally stable (most imidazolium cations), a surface complex with energy transfer is created [22]. In case of thermally unstable ILs cations, e.g., pyridinium and pyrrolidinium, the mechanism occurs by doping with atoms derived from an IL [22]. A completely new one, so far untested in photocatalytic reaction with ILs, is 2,3,5-triphenyltetrazolium chloride [TPTZ][Cl].

In this context, for the first time, the photocatalytic properties of TiO_2_ microspheres with 2,3,5-triphenyltetrazolium chloride IL (organic salt) were investigated. The effects of the IL thermal decomposition during the solvothermal reaction and IL content in the reaction system on photoactivity of TiO_2_ microparticles have been systematically examined. Furthermore, the mechanism of the phenol photocatalytic degradation in the presence of IL-assisted TiO_2_ particles was proposed.

## 2. Materials and Methods

### 2.1. Preparation of the ILs–TiO_2_ Particles

The ILs-assisted TiO_2_ microparticles were synthesized by the procedure developed and optimized in our previous work [8]. Titanium(IV) butoxide (TBOT was provided from Merck, Darmstadt, Germany) used as a precursor of TiO_2_ was dissolved in an absolute ethanol with the purity 99.9% (99.9% of ethanol and 0.1% of water) provided from Avantor Performance Materials Poland S.A., Gliwice, Poland. Then, hydrochloric acid (provided from Avantor Performance Materials Poland S.A., Gliwice, Poland) and distilled water were added. To investigate the effect of the 2,3,5-triphenyltetrazolium chloride ionic liquid (IL) in the reaction mixture on the TiO_2_ properties, the molar ratio of TBOT to IL was variable and ranged from 10:1 to 1:1. IL was provided from Merck, Darmstadt, Germany. The obtained reaction mixture was placed in a Teflon-lined stainless steel autoclave and kept at 180 °C for 1, 4, 12, and 24 h. After the end of the set time, the reactor was cooled down at room temperature. The product was washed with ethanol and deionized water and dried at 50 °C for 12 h, and finally calcined at 200 °C for 2 h. For comparison, reference TiO_2_ was synthesized using the same procedure without addition of IL.

### 2.2. Characterization of ILs–TiO_2_ Particles

Phase purity of the samples was determined by X-ray diffractometer MiniFlex 600 (Rigaku, The Woodlands, TX, USA) equipped with Cu Kα radiation, in the range of 2θ = 20−80°. The average crystallite size was calculated using the Scherrer equation. The morphology and size distribution of the TPTZ_TiO_2_ samples were observed by scanning electron microscope (SEM), JSM-7610F (Jeol, Tokyo, Japan) under high vacuum. The specific surface area and pore size of the photocatalysts (physical adsorption and desorption of nitrogen at 77 K) based on the BET method (determination of the Brunauer–Emmett–Teller isotherm) were measured by Micromeritics Gemini V200 instrument equipped in the VacPrep 061 Degasser (Shimadzu, Kioto, Japan). A Nicolet Evolution 220 UV-Vis spectrophotometer (Thermo, Waltham, MA, USA) was used to obtain the diffuse reflectance UV-VIS absorption spectra of the samples, for which the baseline was performed using barium sulfate. A Flash 2000 CHNS (Thermo Scientific) analyzer was used to determine the elemental composition of the synthesized materials. The X-ray photoelectron spectroscopy (XPS) experiments were performed using a PHI 5000 VersaProbeTM (ULVAC-PHI, Japan/USA) spectrometer with monochromatic Al Kα radiation (hν = 1486.6 eV). The X-ray beam was focused to have a diameter of 100 µm, and the measured area was defined as 250 µm × 250 µm. The high-resolution (HR) XPS spectra were collected by the hemispherical analyzer at a pass energy of 23.5 eV, an energy step size of 0.1 eV, and a photoelectron take off angle of 45° with respect to the surface plane. CasaXPS software (v. 2.3.19PR1.0) was used to evaluate the XPS data. The binding energy (BE) scale of all detected spectra was referenced to the C 1s core level (BE = 284.8 eV).

### 2.3. Photocatalytic Test

To investigate, under the visible light irradiation, the photocatalytic activity of composites of TiO_2_ prepared in the presence of IL, the decomposition rate of phenol (0.21 mmol∙dm^−3^) in an aqueous solution was measured. The mixture of 0.125 g of the photocatalysts in 25 mL of phenol solution was stirred using a magnetic stirrer in a cylindrical reactor with a quartz window. The aeration of suspension (5 dm^3^/h) was kept prior to and during the photocatalytic process. The photoirradiation was provided by a 1000 W Xenon lamp (6271H, Oriel), capable of emitting both the UV and visible light. For all of the measurements, an optical filter was used, with wavelengths λ of >420 nm. During the illumination, aliquots of the aqueous suspension with a volume of 1 cm^3^ were successively sampled. Removal of the fine particles of the photocatalyst was performed by filtering the samples through a 0.2 µm syringe filter. To determine the phenol concentration, the colorimetric method (λ_max_ = 480 nm) was used with the UV-VIS spectrophotometer (Spectro UV-VIS Double Beam UVD-3500, Labmed, Inc., Los Angeles, CA, USA). The blind test (in the absence of photocatalysts or illumination) proceeded the photocatalytic degradation runs. The absence of either the photocatalyst or illumination resulted in lack of phenol degradation.

Controlled photoactivity experiments using different radical scavengers (ammonium oxalate as a scavenger for photogenerated holes, silver nitrate as a scavenger for electrons, benzoquinone as a scavenger for superoxide radical species, and tert-butyl alcohol as a scavenger for hydroxyl radical species) were similarly carried out in the above photocatalytic degradation tests, except that the radical scavengers were added to the reaction system. The scavenger concentration was equal to 0.21 mmol∙dm^−3^. No adsorption of phenol was observed in the presence of photocatalyst in the phenol/scavenger solution and absence of illumination.

The decomposition level of cations was analyzed using. HPLC (Shimadzu, Kioto, Japan) was a system equipped with a diode-array detector SPD-M20A, pump LC-20AD, autosampler SIL 20AHT, column oven CTO-10ASvp and degasser DGU-20A5R. HPLC-grade acetonitrile with addition of 0.025% trifluoroacetic acid (*v*/*v*) and deionized water containing 0.025% TFA (*v*/*v*) were used as a mobile phases A and B, respectively. The separation was carried out with Hypersil Gold aQ column with dimensions: 150 × 4.6; 5 μm (Thermo Scientific) and an isocratic program: 5% of mobile phase A and 95% of phase B at room temperature. The flow rate was 1 mL∙min^−1^, and the elution profiles were monitored at 205 nm or 258 nm. Each sample (before and after solvothermal reaction) was measured in triplicate.

The decomposition level of cations was calculated as:

ηIL (%) = 100 ∗ C_0_ − (C/C_0_) (1)

where C_0_ is the initial concentrations of cations of ILs; C is the concentrations of cations of ILs after the solvothermal reaction.

The experiments of 2,3,5-triphenyltetrazolium cation thermal decomposition during the different reaction times (4, 12, and 24 h) were done for the lowest IL concentration (the IL:TBOT molar ratio was 1:10).

## 3. Results and Discussion

**Figure 1:**
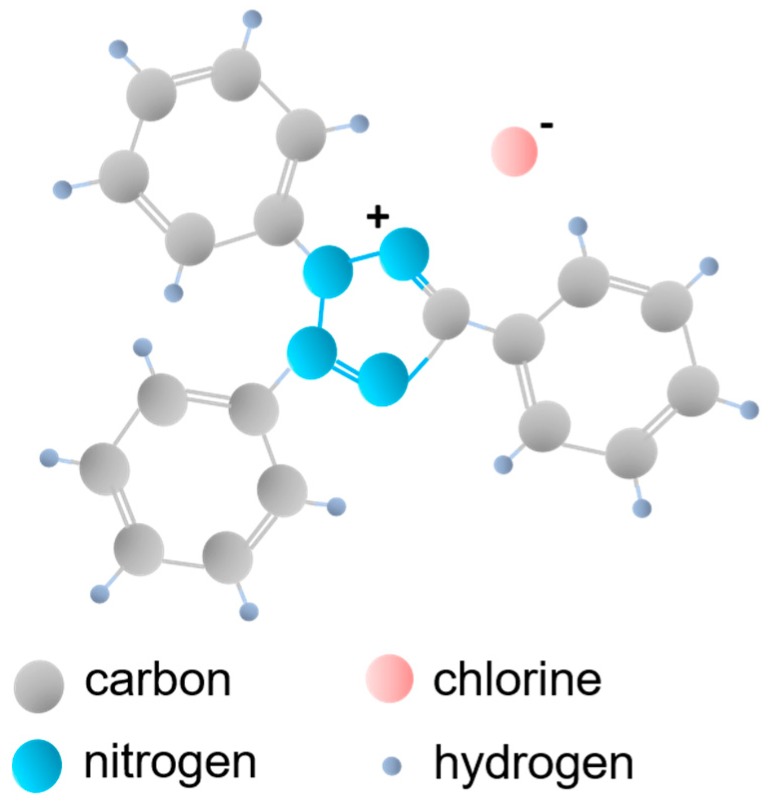
Structure of 2,3,5-triphenyltetrazolium chloride ionic liquid ([TPTZ][Cl]) used for the TPTZ_TiO_2_ microparticles preparation.

To examine the effect of the 2,3,5-triphenyltetrazolium chloride (see Figure 1) on the enhancement of photoactivity under visible irradiation, the TPTZ–TiO_2_ photocatalysts were prepared using various molar ratios of IL to TiO_2_ precursor (IL:TBOT): 1:10; 1:8; 1:5; 1:3; 1:2; and 1:1. At the first stage, we chose the IL:TBOT molar ratio that enabled obtaining the photocatalyst with the highest photoactivity. The next step was to investigate the influence of tetrazolium IL thermal decomposition on the photocatalytic properties of the TiO_2_ particles only for the selected IL:TBOT molar ratio. The surface and optical properties of the selected photocatalysts were then examined. Finally, the mechanism and the role of IL in the photocatalytic activity improvement were discussed.

### 3.1. Photocatalytic Activity

**Figure 2:**
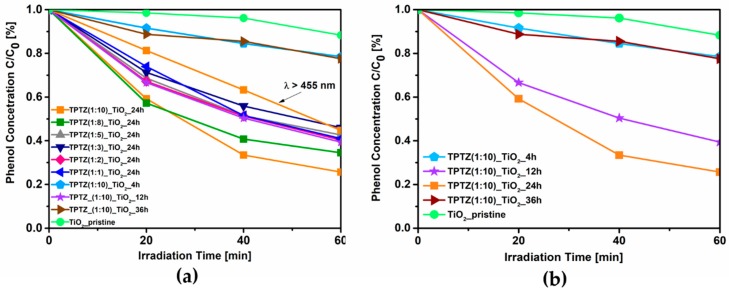
Efficiency of phenol degradation under visible irradiation (λ > 420 nm or λ > 455 nm) in the presence of the TPTZ_TiO_2_ samples prepared in various IL:TBOT molar ratios (**a**) and in different time periods (**b**).

The photocatalytic activity of the TPTZ_TiO_2_ samples and the reference TiO_2_ under visible irradiation (optical filter: λ > 420 nm), evaluated using an aqueous solution of phenol as a model pollutant, is shown in Table 1 and Figure 2. It was found that all samples obtained in the presence of IL revealed higher ability to induce phenol degradation than pristine TiO_2_. Efficiency of phenol degradation increased from 7% up to 74% for pristine and TiO_2_ sample TPTZ(1:10)_TiO_2__24 h, respectively. This is the sample obtained with the lowest IL content (IL/TBOT molar ratio of 1:10) during 24 h of the solvothermal reaction. The highest photocatalytic activity was accompanied by the largest specific surface area (227 m^2^·g^−1^). The more developed specific surface area may provide more active sites and shorten the bulk diffusion length of the charge carriers, thus suppressing bulk recombination [16]. However, further increase of the IL content in the system resulted in decreasing the photoactivity (the IL:TBOT molar ratio ranged from 1:8 to 1:1; see Table 1). Moreover, the efficiency of phenol degradation was also related with the time of the solvothermal synthesis as determined for the TPTZ(1:10)_TiO_2_ sample.

After 1 h reaction time, no product (precipitate) was obtained, and thus photoactivity was not possible to be determined. For the samples obtained during 4 h, 12 h, and 24 h of the solvothermal reaction, the efficiency of the phenol degradation increased from 22% to 61% and 74%, respectively. Therefore, we concluded that elongation of the reaction time is required for precipitation and formation of the structure with large surface area and high photoactivity. These results are in agreement with our previous studies performed with application of 1-butyl-3-methylimidazolium chloride ([BMIM][Cl]) and 1-decyl-3-methylimidazolium chloride ([DMIM][Cl]) ILs [8]. However, further elongation of the synthesis time up to 36 h exerted an opposite effect and photoactivity dropped to 23%. We could conclude that the solvothermal synthesis belongs to the simplest methods of the particles formation, which enable controling properties of the material; however, relatively long reaction time is required.

To elucidate the mechanism of TiO_2_ photoexcitation, an additional experiment with using optical filter with wavelengths λ of >455 nm was also performed for the sample that demonstrated the highest photoactivity (TPTZ(1:10)_TiO_2__24 h). In our previous paper, we revealed that, for the photocatalytic reaction induced by TiO_2_ modified with 1-butyl-3-methylimidazolium bromide ([BMIM][Br]) mainly, the wavelength of 448 nm was used [7]. However, in this experiment, the amount of phenol that was degraded after 60 min of irradiation decreased only to 55% indicating that not only wavelength region 420–455 nm but also longer wavelengths are effectively used in this specific reaction.

### 3.2. Structure, Morphology, and Absorption Properties

In order to explore the effect of time on the reaction yield, size, and shape of the TiO_2_ microparticles, further experiments were carried out for the samples formed with an IL:TBOT molar ratio of 1:10 at various time periods: 1, 4, 12, and 24 h (Table 1). However, as was mentioned above, 1 h reaction time was not sufficient for the effective TiO_2_ nucleation and crystal growth. In this case, no product was achieved at the bottom of the Teflon-lined autoclave. Increasing the reaction time to 4 h promoted formation of the product; however, the reaction yield was relatively low, only 30%. Prolongation of the thermal treatment to 12 and 24 h facilitated the TiO_2_ particles formation which was observed as reaction yields were increased to 45% and 94%, respectively. Higher reaction yield was also accompanied by the rise in the above-mentioned photocatalytic activity and the specific surface area, as shown in Table 1 and Figure 2. In this regard, the highest photoactivity, reaction yield, and BET surface area were obtained for sample TPTZ(1:10)_TiO_2__24 h (reaction yield: 94%, efficiency of phenol degradation: 74%).

**Figure 3:**
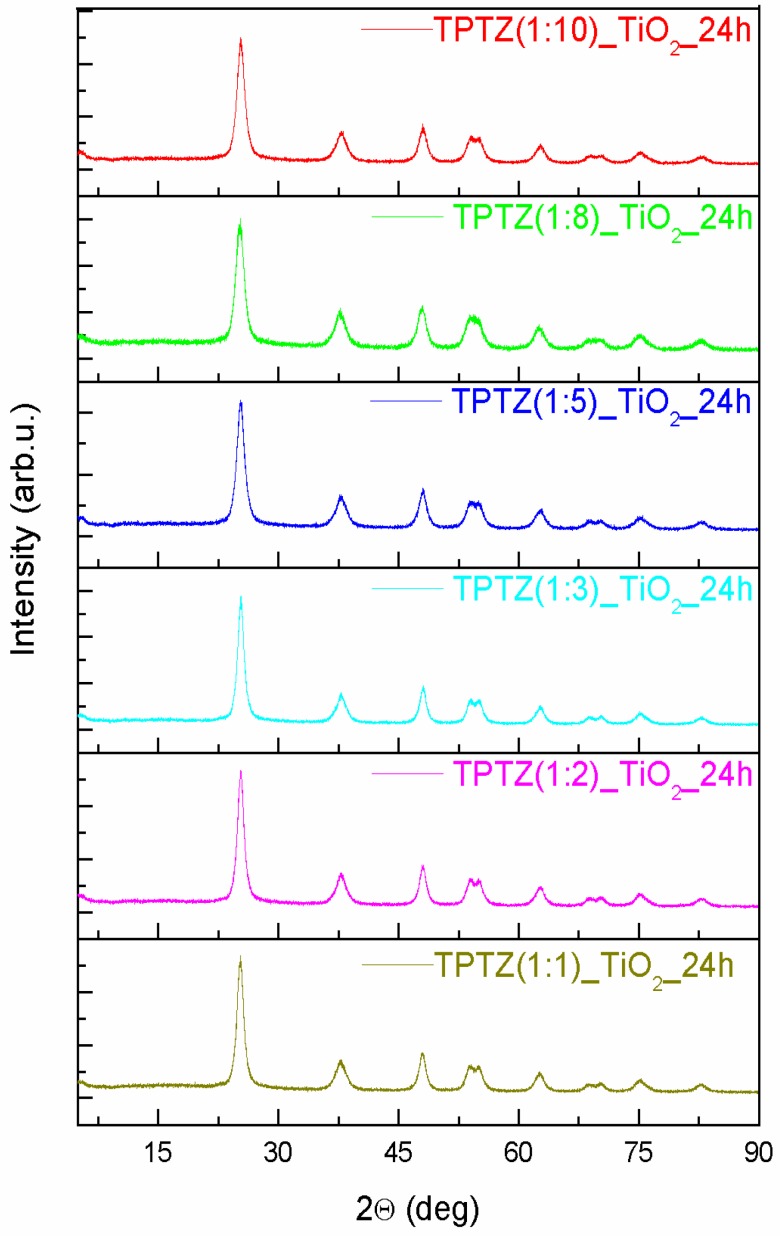
The XRD patterns of the TiO_2_ samples prepared by the ionic liquid (2,3,5-triphenyltetrazolium chloride)-assisted solvothermal method at variable IL contents (the IL:TBOT molar ratio ranged from 1:10 to 1:1).

**Figure 4:**
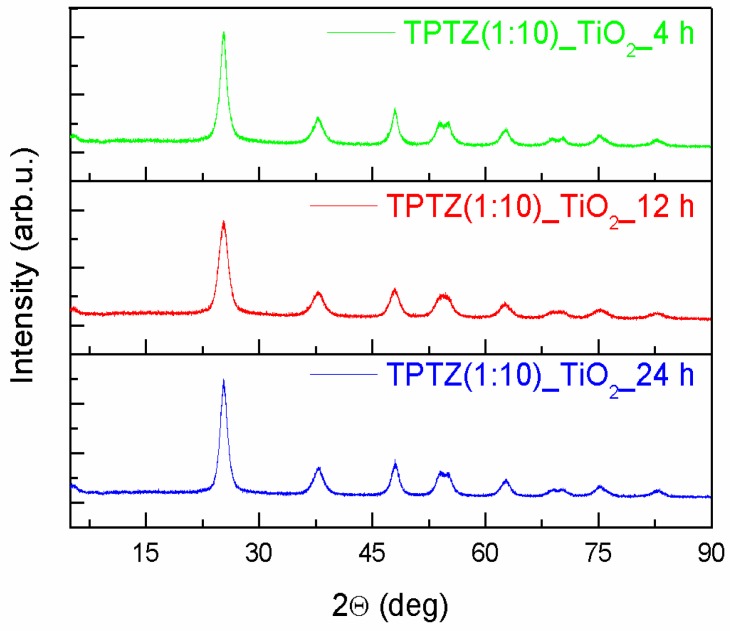
The XRD patterns of the TiO_2_ samples prepared by the 2,3,5-triphenyltetrazolium chloride-assisted solvothermal method at variable time periods (4, 12, and 24 h).

An XRD patterns of the TiO_2_ samples prepared in the presence of the selected tetrazolium IL at various IL contents and preparation time periods are presented in Figure 3; Figure 4. All diffraction peaks look similar and confirmed the complete formation of the anatase phase (JCPDS card No. 89-4921). The anatase phase was represented by (101), (004), (200), (105), (211), (204), (116), (220), (215), and (224) crystal planes. All Bragg reflections were also well indexed to the tetragonal structure (I 41/a m d, s.g. # 141). The most intense peak for anatase (particularly the full width at half maximum), being the one corresponding to the (101) plane detected near 25.3°, was used to quantify the crystallite sizes of the TPTZ_TiO_2_ samples. The mean crystallite sizes, estimated using the Scherrer equation, are presented in Table 2. The lattice parameters for the TPTZ_TiO_2_ samples are also gathered in Table 2.

The Bragg reflections were observed to be broad, indicating that crystallites are small being in the range between 5.7 and 8.8 nm for TPTZ_TiO_2_ prepared for various IL contents. It seems that size of the crystallites increased with increasing of IL amount taken for synthesis; however, the correlation is not unequivocal. Nevertheless, the sample with the highest photocatalytic activity, which is TPTZ(1:10)_TiO_2__24 h, was composed of the smallest crystallites (with diameter 5.7 nm), which justifies the largest BET surface area. Longer time of the solvothermal reaction resulted in a decrease of the size of the crystallites forming the particles, explaining the relation detected for the BET surface area (Table 1).

**Figure 5:**
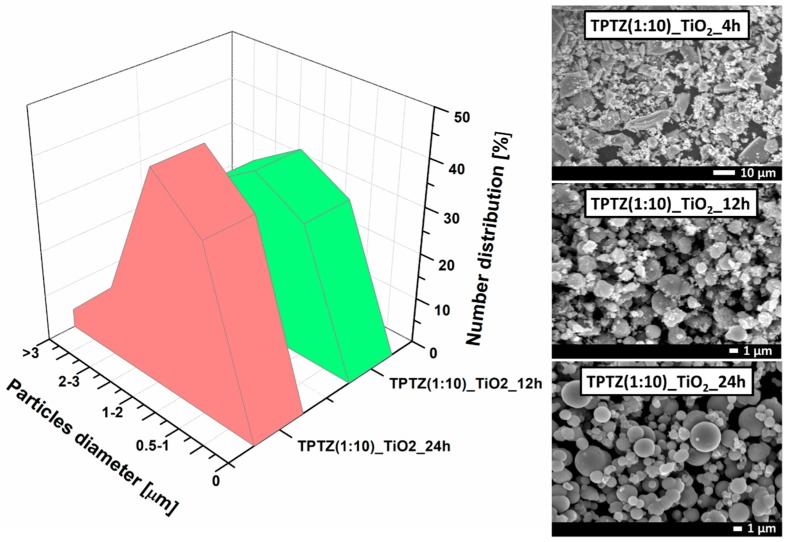
SEM images and particle size distribution of TiO_2_ obtained by the solvothermal method in the presence of [TPTZ][Cl] ionic liquid after 4, 12 and 24 h.

Morphology of the TiO_2_ microparticles obtained in the presence of [TPTZ][Cl] IL was determined using SEM (Figure 5). It was found that the TiO_2_ particles prepared during 4 h of the solvothermal treatment were poorly formed and had an extremely irregular shape. As the reaction time was extended, the particles showed a more spherical shape. After 12 h of the solvothermal treatment, the spheres with a rough surface were detected by SEM. Finally, 24-h synthesis time provided particles with a regular shape and a smooth surface. In this regard, an increased contact time favors TiO_2_ nanocrystals formation and their greater aggregation, allowing time to control the particles growth. The results may indicate that 2,3,5-triphenyltetrazolium chloride as the IL with the large, spatial structure may hinder formation of the spherical particles after 4 h of solvothermal reaction. As reaction time increased to 12 or 24 h, the IL decomposition was higher so steric hindrance was also lower, which further promoted the spheres formation. Therefore, IL played a role of the structuring agent, controlling particles growth, size, and shape during the solvothermal reaction. IL probably played also a role of the factor stabilizing the suspension.

The analysis of the particle size distribution, made by counting the diameters of at least one hundred particles, showed that during 12 and 24 h of the synthesis time microstructures in a range from 1 to 5 μm were obtained. Estimation of the particle size distribution for the sample prepared at the shortest reaction time (TPTZ(1:10)_TiO_2__4 h) was not possible due to the poorly formed particles. In case of the sample synthesized during 12 h of the solvothermal reaction, the particle size distribution ranged from 0.5 to 3 µm. The TPTZ(1:10)_TiO_2__24 h sample was more uniform and consisted mainly of the particles with the size ranging from 0.5 to 2 µm, with the highest contribution of the 1–2 µm structures—45% (Figure 5).

It is worth mentioning that, in comparison with the nanoparticles, the TiO_2_ microparticles offer a highly convenient and flexible system for practical applications (in heterogeneous photocatalysis and other applications). They can be easier separated, collected, and recycled at the end of the process.

**Figure 6:**
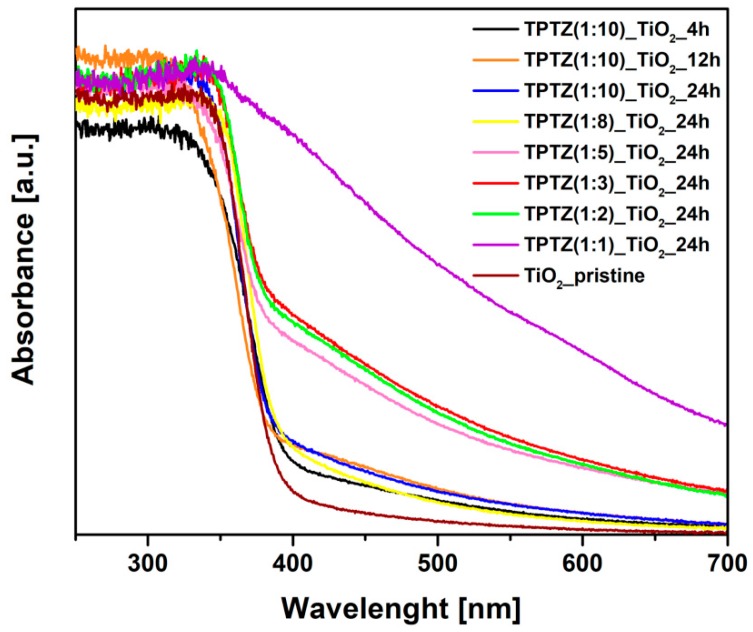
Time-dependent and IL content-dependent changes in the UV-VIS absorption of TPTZ_TiO_2_ microparticles.

**Figure 7:**
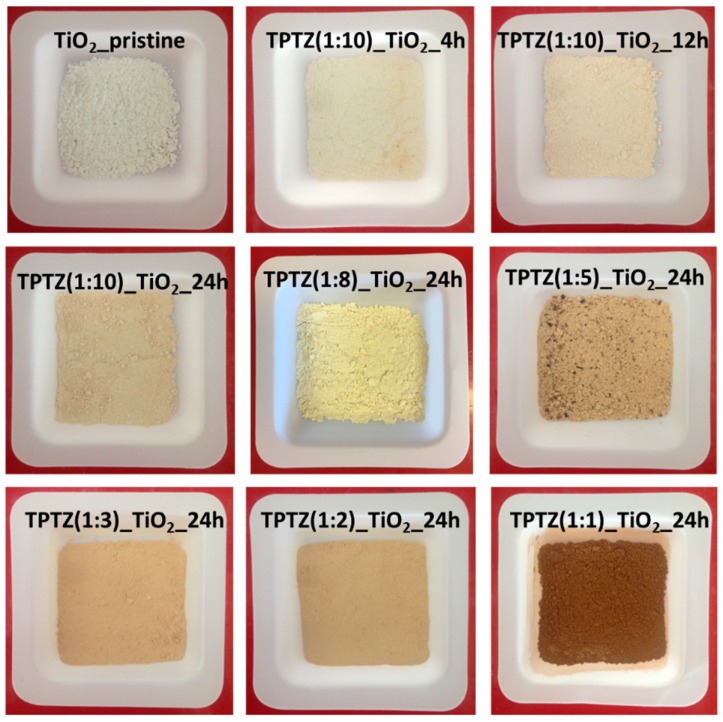
Colors of the samples of pristine TiO_2_ and TiO_2_ obtained using various molar ratios of [TPTZ][Cl] to TBOT in various times of the solvothermal process.

The UV-VIS absorption spectra determined for the photocatalysts obtained in the presence of [TPTZ][Cl] were shown in Figure 6. An absorption shift in the 400–700 nm range was clearly observed for all samples prepared in the presence of IL when compared with pristine TiO_2_, confirming changes in the titania structure. In general, the higher IL amount used for synthesis, the higher enhancement of the absorption in the visible range. Increased absorption was accompanied with a change in color of the samples from white pristine TiO_2_ through beige up to brown TPTZ_TiO_2_ photocatalysts prepared in the presence of the highest amount of IL. Higher content of IL in the reaction system caused a darker color of the photocatalysts (Figure 7). Nevertheless, color of the photocatalyst was not an indicator of the photocatalytic activity. These parameters were inversely dependent. The results also showed that elongation of the reaction time had a little effect on the optical properties. The samples obtained during 12 and 24 h of the reaction time revealed higher absorption in comparison with the photocatalysts prepared at the shorter reaction time.

### 3.3. Surface Composition

Chemical characterization in regard to the surface composition of the ILs–TiO_2_ samples was performed using XPS and FTIR analysis. The elemental surface composition and chemical characteristics of the elements detected at the surface region of these samples are presented in Table 3; Table 4, respectively. The C 1s, N 1s and Cl 2p XPS spectra revealed the modification of the photocatalysts with IL. For TiO_2_ samples prepared at the same period of 24 h, we observe the systematic decrease of carbon and nitrogen content with a decreased amount of IL used for the IL-assisted preparation route (Table 3). The N 1s spectra recorded for TPTZ(1:1)_TiO_2__24 h and TPTZ(1:2)_TiO_2__24 h exhibited two states at BEs of 400.0 eV and 401.5 eV (Table 4). These are samples prepared in a presence of the highest IL content. The first one is assigned to C-N bond [30,31] and second to positively charged N species. The TPTZ(1:3)_TiO_2__24 h and TPTZ(1:5)_TiO_2__24 h samples are characterized only by one nitrogen state at 400 eV. Finally, for the TPTZ(1:8)_TiO_2__24 h and TPTZ(1:10)_TiO_2__24 h samples, an additional nitrogen state appears at BEs of lower than 400 eV, which indicates the Ti–Nx bound formation [32,33]. Detection of this species may indicate doping of the crystalline TiO_2_ structure with N atom originating from decomposition of the tetrazolium cation.

In this regard, the effectiveness of the thermal decomposition of IL during solvothermal reaction was measured by the HPLC method. We found that, after 24 h of the synthesis, tetrazolium-based IL 100% decomposed, indicating that the products may be incorporated into the TiO_2_ structure. Moreover, the TPTZ(1:10)_TiO_2__24 h sample (with the highest photoactivity) was also characterized by the highest amount of surface defects in the form of the Ti^3+^ ions.

**Figure 8:**
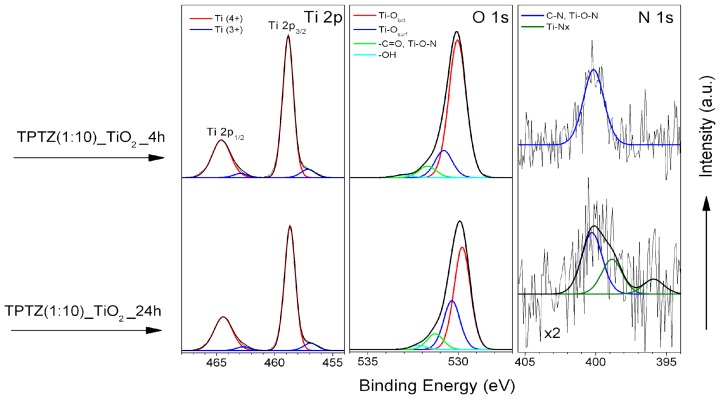
HR XPS spectra of Ti 2p, O 1s and N 1s for TPTZ(1:10)_TiO_2_ after various preparation time periods.

The TiO_2_ samples prepared by using an IL:TBOT molar ratio of 1:10 were also examined after various periods of preparation. The HR XPS spectra of Ti 2p, O 1s and N 1s, recorded for samples prepared after 4 and 24 h of the solvothermal synthesis, are shown in Figure 8. One can see that prolongation of the thermal treatment from 4 to 24 h results in change of both O 1s and N 1s spectra (Figure 8). The oxygen state related to Ti-O_surf_ species becomes more pronounced (see Table 4) and a relative contribution of nitrogen decreases (Table 3). Moreover, an additional nitrogen state appears at BEs of lower than 400 eV [32,33].

**Figure 9:**
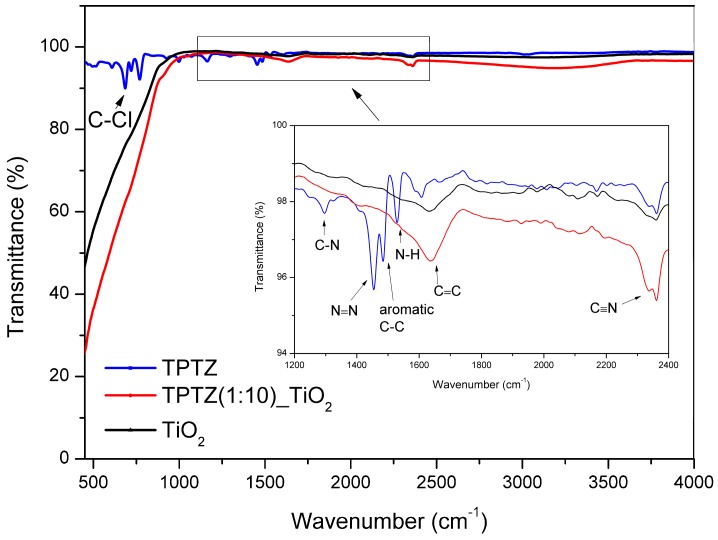
FTIR analysis of pristine IL and TPTZ(1:10)_TiO_2__24 h sample in comparison with that of pristine TiO_2_.

The FTIR analysis was performed for pure IL and the sample TPTZ(1:10)_TiO_2__24 h. The curves presented in Figure 9 correspond to spectra recorded in the wavelength 500–4000 cm^−1^ for TPTZ and TPTZ(1:10)_TiO_2__24 h in comparison with pristine TiO_2_ sample. The strongest signals centered around 690 and 820 cm^−1^ represented characteristic stretching vibrations of C–Cl, which come from interaction between cation and anion in IL. The graph insert wavelength 1200–2400 cm^−1^) allowed distinguishing the characteristic absorption bands for IL, located at 1150, 1470, 1520 and 1560, 1640, and 2320 cm^−1^. However, the FTIR analysis enabled observing only a small enhancement in signal level for TPTZ(1:10)_TiO_2__24 h, which confirms addition of IL. Furthermore, the signals of TPTZ(1:10)_TiO_2__24 h and TiO_2_ show transmittance peaks in the range 500–1000 cm^−1^, which are assigned to the vibrations of Ti–O and Ti–O–Ti bonds and exhibit essentially the same features.

### 3.4. Mechanism of the Photocatalytic Activity of TiO_2__[TPTZ][Cl] Microparticles

The highest photocatalytic activity (74%) among all samples presented in this study was detected for the TPTZ(1:10)_TiO_2__24 h photocatalyst. This product was obtained during 24 h of the solvothermal synthesis by using an IL:TBOT molar ratio of 1:10. This was the lowest IL content in the reaction mixture from the studied range. Detection of the highest efficiency of phenol degradation for this sample was surprising, taking into account our previous studies, where usually increased IL content in the reaction environment resulted in the increase of the photoactivity of the final product [22]. This sample was composed of anatase with the smallest crystallites (with diameter 5.7 nm), and thus the highest specific surface area (227 m^2^·g^−1^). The size distribution of this photocatalysts was relatively uniform and ranged from 0.5 to 2 µm, with the highest contribution of the 1–2 µm structures (45%). An absorption shift in the visible irradiation range was clearly observed for this sample when compared with pristine TiO_2_, confirming modification of TiO_2_ with IL. Additionally, the HPLC analysis revealed that, during 24 h of the reaction mixture thermal treatment (180 °C), IL underwent 100% degradation. Surface modification of titania with residual IL decomposition products was confirmed by XPS analysis by the detection of carbon, nitrogen, hydrogen, and chlorine atoms as well as spices in the form of C–N (286.1 ± 0.1eV and 400 ± 0.1eV), N–C=N (289.0 ± 0.1eV), and Ti–O–N (531.7 ± 0.1eV and 400 ± 0.1eV). Moreover, in the TPTZ(1:10)_TiO_2__24 h sample, an additional nitrogen state appears, indicating interaction of N atoms with TiO_2_ deeper sites (formation of Ti−Nx species). The contribution of Ti−Nx species (44.75% of N) in N fraction did not exceed the amount of IL residues at the TiO_2_ surface represented by Ti−O−N fraction (55.25% N). Doping of the titania lattice structure with nitrogen atoms may explain the enhanced photocatalytic activity under visible irradiation and shift in the optical absorption. Nitrogen can be relatively easily introduced into the TiO_2_ structure due to its comparable atomic size and stability. The incorporation of nitrogen into the TiO_2_ lattice (which can be substitutional and interstitial [34]) leads to mixing of the N 2p states with the O 2p states on the top of the valence band (VB) [35] and formation additional substitutional (N 2p) and interstitial (N–O) levels (Figure 9) [36,37,38]. According to Giamello et al. [39]., substitutional nitrogen states lie just above the VB, while interstitial nitrogen states are located higher in the band gap. Excitation of the electron from these high-energy states to the conduction band (CB) is related with the optical absorption edge shift toward the lower energies (visible) in comparison with pristine TiO_2_ (UV). Also Wang et al. [40] explained that the VB position of the N-doped TiO_2_ does not shift upward due to interstitial N-doping in spite of formation of the N 2p surface state. Moreover, nitrogen doping is accompanied by oxygen vacancy formation due to the large decrease in the formation energy (from 4.2 to 0.6 eV). Indeed, the relatively higher amount of Ti^3+^ species was detected by XPS analysis for this photocatalysts (7.53 at. %). The Ti^3+^ states are located below the titania CB (Figure 9). The interaction of oxygen vacancies with nitrogen impurities in the doped TiO_2_ lattice changes carrier transfer-recombination dynamics, shifting the bang gap absorption to the visible range [41] and enhancing photocatalytic capacity of TiO_2_ in the visible light region [35].

**Figure 10:**
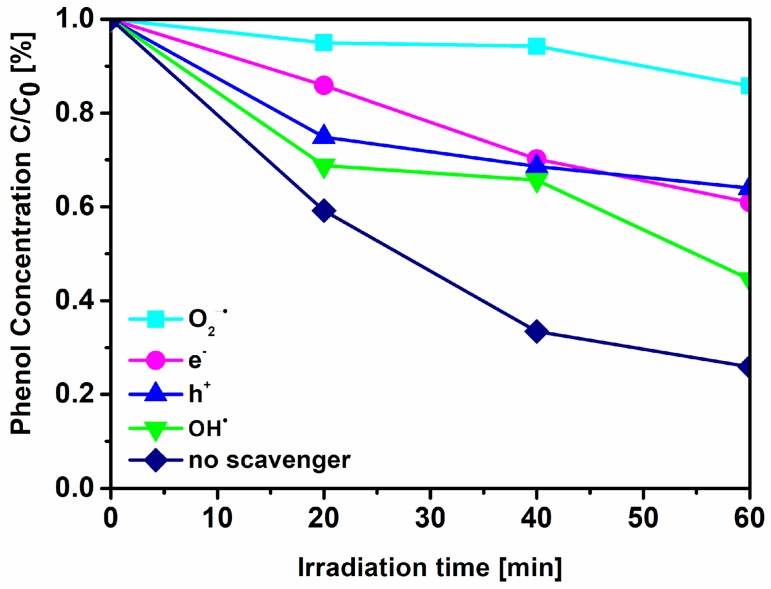
Effects of different scavengers on the effectiveness of phenol degradation under visible irradiation (λ > 420 nm) in the presence of the TPTZ(1:10)_TiO_2__24 h sample.

To assess the role of different reactive oxygen species in the mechanism of phenol decomposition, further studies, employing several specific radical scavengers (ammonium oxalate for h^+^, silver nitrate for e^−^, benzoquinone O_2_^∙−^, and tert-butyl alcohol for ∙OH), were carried out. Benzoquinone, a scavenger of O_2_^∙−^, was found to significantly inhibit the degradation of phenol (Figure 10); however the other reactive oxygen species were also involved in the process. Although O_2_^∙−^ is likely formed in these reaction conditions, ∙OH is much more reactive (redox potential of 2.32 V in comparison with that of 0.89 V for the O_2_^∙−^/H_2_O_2_ couple [42]) and thus may also lead to observed products despite its relatively low concentration. In this regard, under visible irradiation, photogenerated electrons can migrate from the VB, N 2p and N–O energy levels into the CB leaving positive holes. Electrons are involved in reduction of oxygen providing superoxide radicals, while positive holes take part in generation of ∙OH radicals. These reactive species are mainly responsible for the phenol degradation.

To summarize, in our previous study by using the imidazolium salts ([BMIM][X], [X]:Cl, Br, OctSO_4_, PF_6_, and [DMIM][Cl] [7,8]), the photocatalytic activity of the IL–TiO_2_ semiconductors was also dependent on the IL composition and IL content in the reaction environment. Generally, a higher IL content used in synthesis (up to a molar ratio of 1:2) resulted in a higher photodegradation efficiency. However, further increase in the amount of IL in the system had the opposite effect resulting from the overloading of the TiO_2_ surface with IL, which limits equal particle condensation. In case of using the tetrazolium chloride IL, the sample with an IL:TBOT molar ratio of 1:10 showed the highest efficiency of phenol degradation. This phenomenon was probably related with high steric hindrance for 2,3,5-triphenyltetrazolium chloride as the IL with the large, spatial structure. Additionally, the sample TPTZ(1:10)_TiO_2__24 h had the largest specific surface area (227 m^2^·g^−1^), which may provide more active sites and shorten the bulk diffusion length of the charge carriers, and finally suppress bulk recombination [16].

In our previous study by using 1-buthyl-3-methymimidazolium bromide salt, the improved TiO_2_ photoactivity under visible irradiation originates from the interaction of the bromide anion and molecular oxygen with the TiO_2_ surface to form a surface complex that provides CT. We also studied ILs containing distinct nitrogen-bearing organic cations (pyridinium, pyrrolidinium, or ammonium), where observed enhanced photocatalytic activity resulting in interaction of nitrogen atoms with deeper sites of TiO_2_ (Ti-Nx) as well as the highest surface defects in a form of Ti^3+^ [22]. In case of using the tetrazolium chloride, the doping of the crystalline TiO_2_ structure with N atoms originating from IL was proved.

Moreover, for all examined samples, both with the addition of imidazolium and tetrazolium ILs, the phenol degradation was realized mainly by radical anions O_2_^∙−^, whereas the contributions of other processes, involving reactions with trapped electrons (e^-^), holes (h+), and hydroxyl radical (∙OH), were limited in the reaction mechanism.

## 4. Conclusions

The effectiveness of the 2,3,5-triphenyltetrazolium chloride IL thermal decomposition on the structure, optical, and visible light-induced photocatalytic properties of TiO_2_ was investigated. It was found that elongation of the reaction time (1, 4, 12, and 24 h) promoted IL decomposition as well as the growth of the TiO_2_ microparticles with regular, spherical shape. This synergistic effect resulted in formation of the photocatalyst with extremely high photoactivity under visible irradiation, namely up to 74% during 60 min. The improved performance of TiO_2_ microparticles (especially the TPTZ(1:10)_TiO_2__24 h sample) was attributed to small crystallite sizes (with diameter 5.7 nm), well-crystalline anatase phase, high specific surface area (227 m^2^·g^−1^) and uniform size distribution (from 0.5 to 2 µm) of the particles. Moreover, incorporation of the nitrogen atoms into the titania matrix (originating from the decomposition of IL) accompanied by oxygen vacancies formation shifted the bang gap absorption to the visible range and may explain the enhanced photocatalytic activity of TiO_2_ under visible irradiation.

## Figures and Tables

**Table 1:** Characterization and photocatalytic activity of the samples TPTZ_TiO_2_.

Sample Label	IL:TBOT Molar Ratio	Crystalline Phase	Specific Surface Area (m^2^·g^−1^)	Pore Volume (cm^3^·g^−1^)	Efficiency of Phenol Degradation under Visible Irradiation (λ > 420 nm) (%)
TiO_2__pristine	-	anatase	184	0.07	7
TPTZ(1:10)_TiO_2__24 h	1:10		227	0.11	74 (55% λ > 455 nm) *
TPTZ(1:8)_TiO_2__24 h	1:8		212	0.10	66
TPTZ(1:5)_TiO_2__24 h	1:5		187	0.09	57
TPTZ(1:3)_TiO_2__24 h	1:3		201	0.10	58
TPTZ(1:2)_TiO_2__24 h	1:2		198	0.10	59
TPTZ(1:1)_TiO_2__24 h	1:1		219	0.09	59
TPTZ(**1:10**)_TiO_2__1 h	1:10	No precipitate (product) on the bottom of the Teflon-lined autoclave was obtained.			
TPTZ(**1:10**)_TiO_2__4 h	1:10	anatase	185	0.08	22
TPTZ(**1:10**)_TiO_2__12 h	1:10		191	0.09	61
TPTZ(1:10)_TiO_2__36 h	1:10		165	0.08	23

* Photoactivity of this sample was 55% when optical filter with wavelength λ of >455 nm instead of 420 nm was used.

**Table 2:** Structurally refined data from the XRD measurements of the TPTZ_TiO_2_ microparticles.

Sample	a = b (Å)	c (Å)	V (nm^3^)	Crystallite Size (Å)
TiO_2__pristine				
TPTZ(**1:10**)_TiO_2__4 h	3.7768(3)	9.5470(6)	136.18	74
TPTZ(**1:10**)_TiO_2__12 h	3.7963(4)	9.5095(5)	137.05	71
TPTZ(**1:10**)_TiO_2__24 h	3.7913(3)	9.4922(0)	136.44	57
TPTZ(1:8)_TiO_2__24 h	3.7860(2)	9.5474(2)	136.85	66
TPTZ(1:5)_TiO_2__24 h	3.7823(5)	9.5261(2)	136.28	59
TPTZ(1:3)_TiO_2__24 h	3.7814(7)	9.4890(6)	135.68	81
TPTZ(1:2)_TiO_2__24 h	3.7844(0)	9.5254(4)	136.42	60
TPTZ(1:1)_TiO_2__24 h	3.7890(1)	9.5190(0)	136.66	88

**Table 3:** Elemental composition (in at. %) in the surface layer of [TPTZ][Cl]-modified TiO_2_ particles, evaluated by XPS analysis.

Sample	Elemental Composition (at. %)							
	Ti	O	C	N	Cl	C/N	Cl/N	N/Ti
TiO_2_	29.44	66.27	4.14	-	-	-	-	-
TPTZ(1:1)_TiO_2__24 h	19.15	45.59	32.50	2.63	0.15	12.3	0.057	0.1373
TPTZ(1:2)_TiO_2__24 h	21.01	54.78	23.01	1.86	0.19	12.4	0.102	0.0885
TPTZ(1:3)_TiO_2__24 h	23.59	61.30	14.43	0.64	0.04	22.5	0.063	0.0271
TPTZ(1:5)_TiO_2__24 h	23.54	56.53	19.36	0.56	0.11	34.6	0.196	0.0238
TPTZ(1:8)_TiO_2__24 h	25.18	64.64	9.64	0.38	0.15	25.4	0.395	0.0151
TPTZ(1:10)_TiO_2__24 h	24.36	62.12	13.20	0.17	0.14	77.6	0.824	0.0070
TPTZ(1:10)_TiO_2__4 h	24.84	60.56	12.38	0.24	1.97	51.6	8.208	0.0097

**Table 4:** Chemical characteristics of titanium, oxygen, carbon, and nitrogen states in the surface layer of [TPTZ][Cl] IL-modified TiO_2_ particles, evaluated by XPS analysis.

Sample	Ti 2p_3/2_ Fractions (%)		O1s Fractions (%)				C1s Fractions (%)			N1s Fractions		
	Ti(4+) 458.8 ± 0.2 eV	Ti(3+) 457.3 ± 0.1 eV	Ti-O_latt_ 530.0 ± 0.1 eV	Ti-O_surf_ 530.7 ± 0.1 eV	–C=O, Ti-O-N, 531.7 ± 0.1 eV	–OH 532.5 ± 0.1 eV	“A” C–C 284.8 eV	“B” C–O, C–Cl C–N 286.1 ± 0.1 eV	“C” –C=O, N–C=N 289.0 ± 0.1 eV	N+ 401.5 ± 0.1 eV	C–N Ti–O–N 400 ± 0.1 eV	Ti-Nx 396.1–398.9 eV
TPTZ(1:1)_TiO_2__24 h	96.24	3.76	84.03	8.67	5.68	1.63	81.94	15.92	2.14	15.89	84.11	0
TPTZ(1:2)_TiO_2__24 h	96.39	3.61	77.51	10.60	9.43	2.46	89.44	7.34	3.22	9.50	90.50	0
TPTZ(1:3)_TiO_2__24 h	93.71	6.29	65.45	24.65	7.93	1.97	73.12	25.09	1.79	0	100	0
TPTZ(1:5)_TiO_2__24 h	93.58	6.42	65.83	23.53	8.03	2.61	74.63	21.24	4.13	0	100	0
TPTZ(1:8)_TiO_2__24 h	96.46	3.54	74.47	16.44	5.92	3.17	70.56	13.72	15.72	0	86.41	13.59
TPTZ(1:10)_TiO_2__24 h	92.47	7.53	59.74	28.51	9.26	2.49	66.34	29.84	3.82	0	55.25	44.75
TPTZ(1:10)_TiO_2__4 h	93.74	6.26	71.14	20.35	6.84	1.67	72.17	24.73	3.10	0	100	0
